# Effects of exposure to 2100 MHz GSM-like radiofrequency electromagnetic field on auditory system of rats^☆^

**DOI:** 10.1016/j.bjorl.2016.10.004

**Published:** 2016-11-05

**Authors:** Metin Çeliker, Abdulkadir Özgür, Levent Tümkaya, Suat Terzi, Mustafa Yılmaz, Yıldıray Kalkan, Ender Erdoğan

**Affiliations:** a Recep Tayyip Erdogan University, Research and Training Hospital, Department of Otorhinolaryngology, Rize, Turkey; b Recep Tayyip Erdogan University, Medical Faculty, Department of Otorhinolaryngology, Rize, Turkey; c Recep Tayyip Erdogan University, Medical Faculty, Department of Histology and Embryology, Rize, Turkey; d Selçuk University, Medical Faculty, Department of Histology and Embryology, Konya, Turkey

**Keywords:** Cochlear nuclei, Neuronal degeneration, Electromagnetic radiation, Núcleo coclear, Degeneração neuronal, Radiação eletromagnética

## Abstract

**Introduction:**

The use of mobile phones has become widespread in recent years. Although beneficial from the communication viewpoint, the electromagnetic fields generated by mobile phones may cause unwanted biological changes in the human body.

**Objective:**

In this study, we aimed to evaluate the effects of 2100 MHz Global System for Mobile communication (GSM-like) electromagnetic field, generated by an electromagnetic fields generator, on the auditory system of rats by using electrophysiological, histopathologic and immunohistochemical methods.

**Methods:**

Fourteen adult Wistar albino rats were included in the study. The rats were divided randomly into two groups of seven rats each. The study group was exposed continuously for 30 days to a 2100 MHz electromagnetic fields with a signal level (power) of 5.4 dBm (3.47 mW) to simulate the talk mode on a mobile phone. The control group was not exposed to the aforementioned electromagnetic fields. After 30 days, the Auditory Brainstem Responses of both groups were recorded and the rats were sacrificed. The cochlear nuclei were evaluated by histopathologic and immunohistochemical methods.

**Results:**

The Auditory Brainstem Responses records of the two groups did not differ significantly. The histopathologic analysis showed increased degeneration signs in the study group (*p* = 0.007). In addition, immunohistochemical analysis revealed increased apoptotic index in the study group compared to that in the control group (*p* = 0.002).

**Conclusion:**

The results support that long-term exposure to a GSM-like 2100 MHz electromagnetic fields causes an increase in neuronal degeneration and apoptosis in the auditory system.

## Resumo

**Introdução:**

O uso de telefones celulares tornou-se generalizado nos últimos anos. Embora benéfico do ponto de vista da comunicação, os campos eletromagnéticos gerados por celulares pode causar alterações biológicas indesejáveis no corpo humano.

**Objetivo:**

Nesse estudo, o objetivo foi avaliar os efeitos do campo eletromagnético na frequência de 2.100 MHz, similar à modulação do Sistema Global para Comunicações Móveis, produzido por um gerador de campo eletromagnético, sobre o sistema auditivo de ratos usando os métodos eletrofisiológico, histopatológico e imunohistoquímico.

**Método:**

Foram incluídos no estudo catorze adultos ratos albinos Wistar. Os ratos foram divididos aleatoriamente em dois grupos de sete animais cada. O grupo de estudo foi exposto continuamente por 30 dias a um campo eletromagnético em 2100 MHz com um nível de sinal (potência) de 5,4 dBm (3,47 miliwatts) para simular o modo de conversação em um celular. O grupo controle não foi exposto ao campo eletromagnético acima mencionado. Após 30 dias, o potencial evocado auditivo de tronco encefálico de ambos os grupos foi gravado e os ratos foram sacrificados. Os núcleos cocleares foram avaliados pelos métodos histopatológico e imunohistoquímico.

**Resultados:**

Os registros do potencial evocado auditivo de tronco encefálico dos dois grupos não diferiram significativamente. A análise histopatológica mostrou aumento dos sinais de degeneração no grupo de estudo (*p* = 0,007). Além disso, a análise imuno-histoquímica revelou aumento do índice de apoptose no grupo de estudo em comparação com o grupo controle (*p* = 0,002).

**Conclusão:**

Os resultados confirmam que a exposição a longo prazo a um campo eletromagnético em 2100 MHz similar à modulação do sistema global para comunicações móveis causa um aumento na degeneração neuronal e apoptose no sistema auditivo.

## Introduction

The use of mobile phones has become widespread in recent years. Although beneficial from the communication viewpoint, the electromagnetic fields (EMF) generated by mobile phones may cause unwanted biological changes in the human body.^1^, ^2^

The operating frequencies of wireless devices range from 30 kHz to 300 GHz. Devices operating in this frequency range produce an effect area called Radiofrequency Electromagnetic Field (RF-EMF).^3^ Mobile phones operate in the 800–3500 MHz frequency range, and third-generation (3G) mobile phones primarily use the 2100 MHz frequency.^4^ The EMF generated by these devices and base stations that connect them has emerged as a growing public health issue.^1^ In a consensus statement published by the World Health Organization International Agency for Research on Cancer Monograph Working Group in 2011, RF-EMF was accepted as a possible carcinogen for humans after evaluating human and experimental studies on the impact of RF-EMF.^3^ Two large multicenter case-control studies investigated the relationship between brain tumors and mobile phone use. One of these studies found a significant association between mobile phone use and the occurrence of malignant brain tumor.^5^ In contrast, the other study showed that 10 years of mobile phone use did not increase the risk of acoustic neuroma significantly, but it was emphasized that the follow-up period was insufficient for acoustic neuroma, a slowly growing tumor.^6^

Studies investigating the effects of mobile phone use on the auditory system have usually focused on cochlear hearing loss, and otoacoustic emission studies have been preferred for evaluations. Several of these studies found no significant effect of RF-EMF on the auditory system, whereas other studies had minor findings such as a temporary threshold shift or decreased otoacoustic emission responses.^2^, ^7^, ^8^ Retrocochlear pathology is the expected biological damage due to RF-EMF exposure. However, otoacoustic emission testing is used to evaluate the cochlea. Therefore, to evaluate the effects of RF-EMF on the auditory system, the retrocochlear area should be investigated with tests, and to demonstrate biological damage, these tests should be supported by histopathological findings.^9^

In this study, we aim to evaluate the effects of 2100 MHz Global System for Mobile communication (GSM)-like EMF, generated by an EMF generator, on the auditory system of rats by using electrophysiological, histopathologic, and immunohistochemical methods.

## Methods

Following approval by the Local Ethics Committee for Animal Experiments (n° 2014/50), the study was carried out according to the principles of animal research regulations.

### Animals

Fourteen healthy, male, adult Wistar albino rats ranging in weight from 250–280 g were included in the study. During the study, the rats were kept at the experimental animal unit in 12 h of light and 12 h of darkness each day at a room temperature of 22 ± 3 °C and humidity of 55–60%. The animals were allowed to consume unlimited food and tap water, ad libitum. At every stage of the study, the external auditory canal and the tympanic membranes of the rats were examined otoscopically to exclude other factors that may influence the test results, such as signs of infection, tympanic membrane perforation, or cerumen. The rats were divided into two groups. The RF-EMF group was subjected to electromagnetic waves for 30 days. The control group was not exposed to EMF.

### RF-EMF exposure system

The RF-EMF group was exposed to a continuous EMF produced by an EMF generator (Anritsu MG3670B, Japan) for 30 days. The generator was adjusted at a signal level (power) of 5.4 dBm (3.47 mW) and a frequency of 2100 MHz to simulate the talk mode on a mobile phone. The EMF was generated through a 15 cm long rod antenna placed below the plastic rat cage and positioned parallel to the cage's short axis. The rats were allowed to move freely at a maximum distance of 20 cm from the antenna because the exposure was planned, long-term, and continuous.

### Measurement of Auditory Brainstem Response

The Auditory Brainstem Responses (ABRs) of the rats were recorded under anesthesia with 45 mg/kg ketamine hydrochloride (Ketalar^®^, Zentiva, İstanbul, Turkey) and 5 mg/kg xylazine hydrochloride applied intraperitoneally to both groups. The ABR recordings were obtained with 50 dB nHL click stimuli, which were applied using insert earphones and subcutaneous needle electrodes. During the tests, the active electrode was placed at the vertex, reference electrode was placed at the ipsilateral mastoid, and ground electrode was placed on the back of the rats. We recorded 500 sweeps for each test and employed a 0.3–3.0 kHz filter. All recording was performed using an Eclipse EP25 (Interacoustics, Denmark). The latency of wave *V* and interpeak wave latency of waves *I*–*V* were analyzed for both ears.

### Histopathologic and immunohistochemical evaluation

The rats were sacrificed under anesthesia with ketamine HCl after the ABRs were recorded and brain tissues were removed completely. The tissues were fixed in 4% freshly prepared and cooled para-formaldehyde solution for 24 h. Then, cochlear nuclei were removed and waited in a mixture of 30% sucrose and 0.1% sodium azide until they reached sucrose saturation. Using Cryostat (Leica CM1900, Leica Microsystems GmbH, Vienna- Austria), 4 μm thick serial sections were fixed on poly-l lysine-coated slides.

The sections were stained with Hematoxylin–Eosin (HE) for histopathologic evaluation. At least five sections from each rat were examined using an Olympus BX51 photo microscope (Olympus, Tokyo, Japan), and digital images were taken. Signs of degeneration, such as the presence of red neurons, vacuolization, cellular degeneration, edema, or pyknotic cells, were scored separately by two histopathologists blinded to group information. Each rat was graded on a five point scale from 0 to 4+ for signs of degeneration.

In addition to degeneration grading, to evaluate neurotoxicity at the cellular level, the cells were marked immunohistochemically by using the Terminal deoxynucleotidyl transferase dUTP nick end labeling (TUNEL) method, which shows DNA breaks by marking the terminal ends of the nucleic acids involved in apoptotic processes. Sections marked with the TUNEL method were examined by light microscopy. Digital micro-photographs were taken by a photo microscope from at least five different areas. The percentage of apoptotic cells was determined by counting whole cells without distinguishing between neurons and glial cells. The apoptotic index of each rat for different regions was calculated as Total Number of Apoptotic Cells/100. The average apoptotic index of each rat was calculated by averaging the obtained results.

### Statistical analysis

Data were analyzed with SPSS version 15.0 for Windows (SPSS Inc., Chicago, IL, USA). ABR latencies were compared by performing the Mann–Whitney *U* test. Degeneration signs were compared with the chi-square test. A *p*-value less than 0.05 was considered as a significant difference.

## Results

### ABR results

The average measured latency of wave *V* in the control group was 4.54 ± 0.29 ms (4.00–4.93 ms) while that in the study group was 4.72 ± 0.17 ms (4.47–4.93 ms). Similarly, the average interpeak latency between waves *I*–*V* in the control group was 3.38 ± 0.42 ms (2.53–3.73 ms) while that in the study group was 3.69 ± 0.20 ms (3.47–4.06 ms). Compared to the control group, the mean wave *V* latency of the study group was prolonged by about 0.18 ms, and the interpeak latency of *I*–*V* was 0.31 ms. However, this extension was not statistically significant (*p* > 0.05).

### Histopathologic and immunohistochemical evaluation results

**Figure 1:**
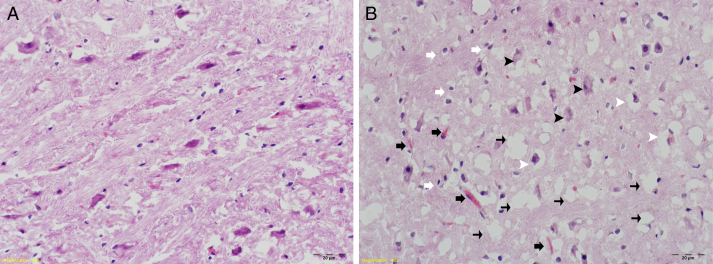
Histomorphological examination H&E staining (40×); (A) control group: tissue integrity, general appearance of cells is natural, no changes in neuron size. (B) RF-EMF group: degeneration of neurons in the ventral cochlear nucleus, degradation (black arrowheads), in addition to some decrease in neuron size, shrunken pyknotic cells (white arrowheads). Increased numbers of glial cells (white arrows) and areas with increased vascularization (black arrows). Intense vacuolization in tissue (thin black arrows) and edematous areas.

The histopathologic degeneration grade findings for both groups are summarized in Table 1. Grading scores for degeneration were significantly higher in the RF-EMF group (*p* = 0.007). In addition, increased numbers of glial cells and vascularization were observed in the same group (Fig. 1).

**Table 1:** Grading scores for degeneration in groups.

	Grading scores for degeneration *n* (%)				
	0	1+	2+	3+	4+
Control group (*n* = 7)	6 (85.7)	1 (14.3)			
RF-EMF group (*n* = 7)			1(14.3)	4 (57.1)	2 (28.6%)

**Figure 2:**
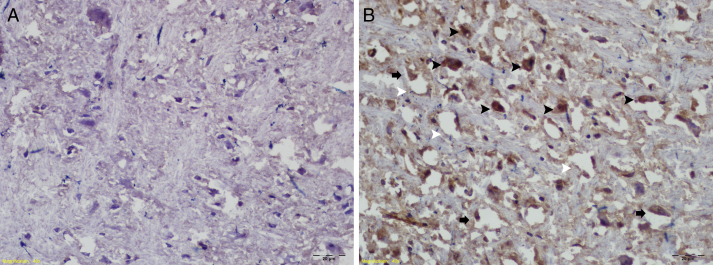
TUNEL staining results; (A) control group; (B) study group: apoptotic cells (black arrowheads), pyknotic neurons (black arrows), diffuse vacuolated areas (white arrowheads).

Immunohistochemical examinations with the TUNEL method showed pyknotic neurons, increased apoptosis, and diffuse vacuolated areas in the RF-EMF group (Fig. 2).

**Figure 3:**
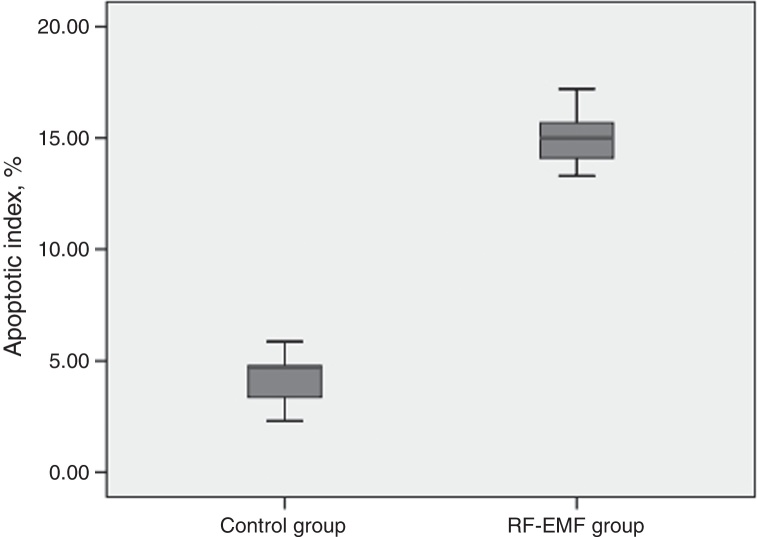
Graph of apoptotic index for groups.

A comparison of the apoptotic indexes of the groups revealed that the index of the study group was significantly higher than that of the control group. The average apoptotic index was 14.78% in the RF-EMF group while it was 4.17% in the control group (Fig. 3).

## Discussion

This is the first study showing the histopathological and electrophysiological effects of 2100 MHz GSM-like EMF on the auditory system. In a previous study performed in our center, degeneration of cochlear nuclei was observed after chronic exposure to EMF at 1800 MHz.^10^ Based on a review of the literature and to our knowledge, no such study has been conducted with a 2100 MHz EMF. The results obtained in this study show that in rats, chronic exposure to 2100 MHz GSM-like EMF leads to cellular degeneration in the cochlear nuclei with increased apoptosis and statistically insignificant prolongation of ABR wave latencies.

With rapid increase in the use of mobile phones worldwide, the EMF generated by these devices and base stations that connect these devices has emerged as a major public health problem. Although RF-EMF is accepted as a possible carcinogen by international working groups, it has been emphasized in the report of Advisory Group on Non-ionizing Radiation (AGNIR) that no convincing evidence about the genotoxicity and carcinogenicity of RF-EMF has been revealed in various studies.^5^, ^11^ Temporary symptoms resulting from increased duration of daily mobile phone usage such as headaches, lack of concentration, sleep disorders, and increase in temperature around the ear were shown in earlier studies.^12^ However, case-control and cross-sectional studies evaluating the long-term effects RF-EMF found different results. An important point in these studies was that for determining whether exposure to RF-EMF results in the occurrence of tumors such as acoustic neuromas, the follow-up period should be adequately long.^5^, ^6^ The data obtained in the present study shows that chronic exposure to RF-EMF could cause degeneration in the cochlear nuclei of rats. In a previous study performed in our center, some degree of degeneration in the cochlear nuclei in rats was observed upon histopathological and immunohistochemical examinations after chronic exposure to EMF at 1800 MHz.^10^ In addition to histopathological degeneration and different from the previous study, increased apoptotic index of the cochlear nuclei was observed using immunohistochemical TUNEL assay in the present study. We believe that this increase in the apoptotic index is likely to be an indicator of the genotoxic and carcinogenic effects of RF-EMF. Given that our RF-EMF exposure system was continuous and long-term, it was not an exact simulation of daily mobile phone use. Therefore, based on our results, one cannot say that the RF-EMF generated by mobile phone use is genotoxic and carcinogenic for humans.

With the widespread use of mobile phones, the first experimental studies evaluating the effects of RF-EMF on the auditory system preferred the use of otoacoustic emissions tests more often. These test results were statistically non-significant.^13^, ^14^ The main limitation of these studies was the inability to evaluate retrocochlear damage due to their use of otoacoustic emissions. Moreover, ABR studies were performed to evaluate the expected retrocochlear effects of RF-EMF in humans. However, there was no change in terms of wave latencies.^15^, ^16^ According to the data we obtained, prolonged wave latencies were identified with ABR results in the study group compared to the control group, but these findings were not statistically significant. Although histopathological and immunochemical analyses showed significant degeneration in the cochlear nuclei, this did not cause significant prolongation of ABR wave latency. This finding could be attributed to damages that did not disturb stimulus transmission in the cochlear nuclei. However, lack of significant prolongation in ABR wave latencies does not indicate that transmission is exactly intact.

One limitation of this study is that exposure of the rats to EMF was not highly standardized in our RF-EMF exposure system. Several systems designed for EMF exposure have been described in the literature.^17^ However, these systems are unsuitable for long-term EMF application, which was required in our study. Since our EMF exposure regime was long-term and continuous, we designed an experimental system in which the rats could move freely and easily perform daily activities such as eating and drinking. However, the rats were not allowed to move more than 20 cm away from the EMF antenna.

## Conclusion

The data obtained in this study shows that chronic RF-EMF exposure causes degeneration of the cochlear nuclei in rats. An increase in the apoptotic index was determined by immunohistochemical methods in cochlear nuclei as a result of this degeneration. However, there was no statistically significant electrophysiological prolongation in ABR wave latencies. These findings support the possible genotoxic and carcinogenic effects of RF-EMF.

## Conflicts of interest

The authors declare no conflicts of interest.
